# Gene co-expression analysis identifies brain regions and cell types involved in migraine pathophysiology: a GWAS-based study using the Allen Human Brain Atlas

**DOI:** 10.1007/s00439-016-1638-x

**Published:** 2016-02-22

**Authors:** Else Eising, Sjoerd M. H. Huisman, Ahmed Mahfouz, Lisanne S. Vijfhuizen, Verneri Anttila, Bendik S. Winsvold, Tobias Kurth, M. Arfan Ikram, Tobias Freilinger, Jaakko Kaprio, Dorret I. Boomsma, Cornelia M. van Duijn, Marjo-Riitta R. Järvelin, John-Anker Zwart, Lydia Quaye, David P. Strachan, Christian Kubisch, Martin Dichgans, George Davey Smith, Kari Stefansson, Aarno Palotie, Daniel I. Chasman, Michel D. Ferrari, Gisela M. Terwindt, Boukje de Vries, Dale R. Nyholt, Boudewijn P. F. Lelieveldt, Arn M. J. M. van den Maagdenberg, Marcel J. T. Reinders

**Affiliations:** Department of Human Genetics, Leiden University Medical Center, 2333 ZC Leiden, The Netherlands; Delft Bioinformatics Lab, Department of Intelligent Systems, Delft University of Technology, 2628 CD Delft, The Netherlands; Division of Image Processing, Department of Radiology, Leiden University Medical Center, 2333 ZA Leiden, The Netherlands; Analytical and Translational Genetics Unit, Department of Medicine, Massachusetts General Hospital and Harvard Medical School, Boston, MA 02114 USA; Program in Medical and Population Genetics, Broad Institute of MIT and Harvard, Cambridge, MA 02142 USA; Stanley Center for Psychiatric Research, Broad Institute of MIT and Harvard, Cambridge, MA 02142 USA; FORMI and Department of Neurology, Oslo University Hospital and University of Oslo, 0424 Oslo, Norway; Institute of Public Health, Charité - Universitätsmedizin Berlin, 10117 Berlin, Germany; Division of Preventive Medicine, Department of Medicine, Brigham and Women’s Hospital, Harvard Medical School, Boston, MA 02215-1204 USA; Department of Epidemiology, Erasmus University Medical Centre, 3015 CE Rotterdam, The Netherlands; Department of Radiology, Erasmus University Medical Centre, 3015 CE Rotterdam, The Netherlands; Department of Neurology, Erasmus University Medical Centre, 3015 CE Rotterdam, The Netherlands; Department of Neurology and Epileptology and Hertie-Institute for Clinical Brain Research, University of Tübingen, 72076 Tübingen, Germany; Institute for Stroke and Dementia Research, Klinikum der Universität München, Ludwig-Maximillians-Universität, 81377 Munich, Germany; Department of Public Health, University of Helsinki, 00014 Helsinki, Finland; Department of Biological Psychology, VU University, 1081 HV Amsterdam, The Netherlands; Department of Epidemiology and Biostatistics, MRC–PHE Centre for Environment and Health, School of Public Health, Imperial College London, London, W2 1PG UK; Center for Life-Course Health Research and Northern Finland Cohort Center, Faculty of Medicine, University of Oulu, P.O. Box 5000, 90014 Oulu, Finland; Biocenter Oulu, University of Oulu, Aapistie 5A, P.O. Box 5000, 90014 Oulu, Finland; Unit of Primary Care, Oulu University Hospital, Kajaanintie 50, 90029 OYS, P.O. Box 20, 90220 Oulu, Finland; Department of Twin Research and Genetic Epidemiology, King’s College London, London, SE1 7EH UK; Population Health Research Institute, St George’s, University of London, London, SW17 0RE UK; Institute of Human Genetics, University Medical Center Hamburg-Eppendorf, 20246 Hamburg, Germany; Munich Cluster for Systems Neurology (SyNergy), 81377 Munich, Germany; Medical Research Council Integrative Epidemiology Unit (IEU), School of Social and Community Medicine, University of Bristol, Bristol, BS8 2PS UK; deCODE Genetics, 101 Reykjavik, Iceland; School of Medicine, University of Iceland, 101 Reykjavik, Iceland; Institute for Molecular Medicine Finland (FIMM), University of Helsinki, 00290 Helsinki, Finland; Department of Neurology, Leiden University Medical Center, 2333 ZA Leiden, The Netherlands; Institute of Health and Biomedical Innovation, Queensland University of Technology, Kelvin Grove, QLD, Brisbane, QLD 4059 Australia; Queensland Institute of Medical Research (QIMR) Berghofer Medical Research Institute, Brisbane, QLD 4006 Australia

## Abstract

Migraine is a common disabling neurovascular brain disorder typically characterised by attacks of severe headache and associated with autonomic and neurological symptoms. Migraine is caused by an interplay of genetic and environmental factors. Genome-wide association studies (GWAS) have identified over a dozen genetic loci associated with migraine. Here, we integrated migraine GWAS data with high-resolution spatial gene expression data of normal adult brains from the Allen Human Brain Atlas to identify specific brain regions and molecular pathways that are possibly involved in migraine pathophysiology. To this end, we used two complementary methods. In GWAS data from 23,285 migraine cases and 95,425 controls, we first studied modules of co-expressed genes that were calculated based on human brain expression data for enrichment of genes that showed association with migraine. Enrichment of a migraine GWAS signal was found for five modules that suggest involvement in migraine pathophysiology of: (i) neurotransmission, protein catabolism and mitochondria in the cortex; (ii) transcription regulation in the cortex and cerebellum; and (iii) oligodendrocytes and mitochondria in subcortical areas. Second, we used the high-confidence genes from the migraine GWAS as a basis to construct local migraine-related co-expression gene networks. Signatures of all brain regions and pathways that were prominent in the first method also surfaced in the second method, thus providing support that these brain regions and pathways are indeed involved in migraine pathophysiology.

**Electronic supplementary material:**

The online version of this article (doi:10.1007/s00439-016-1638-x) contains supplementary material, which is available to authorized users.

## Introduction

Migraine is a common neurovascular brain disorder characterised by attacks of severe, unilateral headache, often accompanied by nausea and phono- and photophobia (Headache Classification Committee 2013). Two main migraine types are distinguished based on the presence or absence of an aura, which consists of transient neurologic symptoms including visual and sensory disturbances that can precede attacks in up to one-third of patients. Migraine is a complex genetic disorder with an estimated heritability of approximately 50 % (Mulder et al. 2003) and thought to be caused by an interplay of multiple genetic variants, each with a small effect size, and environmental factors. Numerous candidate gene association studies have been performed for migraine, however, their value turned out rather low as none could be replicated in a large genome-wide marker dataset of thousands of migraine patients and controls (de Vries et al. 2015). Genome-wide association studies (GWAS) investigating the common forms of migraine have identified 13 disease susceptibility loci (Anttila et al. 2010, 2013; Chasman et al. 2011; Freilinger et al. 2012). These loci identified genes that are involved in glutamatergic neurotransmission (*MTDH*, *LRP1*, *MEF2D*), neuron and synapse development (*MEF2D*, *ASTN2*, *PRDM16*, *FHL5*, *PHACTR1*, *TGFBR2* and *MMP16*), brain vasculature (*PHACTR1*, *TGFBR2*, *C7orf10*), extracellular matrix (*MMP16*, *TSPAN2*, *AJAP1*), and pain-sensing (*TRPM8*). These findings support knowledge that came from investigating disease mechanisms in monogenic migraine-related disorders including familial hemiplegic migraine (FHM), a monogenic subtype of migraine with aura (Ferrari et al. 2015; Tolner et al. 2015). Notably, transgenic knock-in (KI) mouse models that express human pathogenic FHM1 (van den Maagdenberg et al. 2004, 2010) or FHM2 (Leo et al. 2011) mutations revealed increased susceptibility for experimentally induced cortical spreading depression (CSD), the electrophysiological correlate of the migraine aura (Lauritzen 1994), which could be directly linked to increased cortical glutamatergic neurotransmission in FHM1 KI mice (Tottene et al. 2009). Other monogenic disorders in which migraine is prevalent are cerebral autosomal dominant arteriopathy with subcortical infarcts and leukoencephalopathy (CADASIL) and retinal vasculopathy with cerebral leukodystrophy (RVCL) that indicate a role for dysfunction of the brain vasculature in migraine (Tolner et al. 2015). Migraine genes identified by GWAS are primarily identified based on their location near top hits, so true causality of (at least some of) them remains uncertain, which is not different from other disorders. Furthermore, current GWAS top hits explain only a small part of the disease heritability, and, therefore, genes identified in this way reflect only a fraction of the pathways conferring genetic disease risk. Hence, pathway analysis methods that harvest a larger portion of the GWAS data (i.e., not only loci with significant *P* values) may give more valuable insight into disease genetics, as has been tried for other diseases (Atias et al. 2013; Sun 2012).

Commonly used tools to explore disease-associated pathways in GWAS data make use of functional enrichments [MAGENTA Gene Set Enrichment Analysis (Segre et al. 2010)], protein interactions [DAPPLE (Rossin et al. 2011)] or text-mining [GRAIL (Raychaudhuri et al. 2009)], but did not successfully identify overrepresented molecular pathways involved in migraine (Anttila et al. 2013). One explanation why it may be difficult to confidently identify disease pathways from GWAS data is that loci often contain multiple genes, of which only (one or) a subset might influence the trait of interest. Moreover, each of these genes can be expressed in multiple cell types and may have different functions in each of them. We envisaged that gene expression data can be used to preselect genes for functional analysis based on their expression in disease-relevant tissues, thereby increasing the chance of identifying disease-relevant genes and pathways. In addition, gene co-expression analysis can be used to identify genes with similar expression patterns. Previous studies have shown that gene co-expression can infer a wide range of meaningful biological information, e.g., shared gene functions, biological pathways or cell type-specific expression (Kang et al. 2011; Hawrylycz et al. 2012; Grange et al. 2014).

Gene co-expression analysis has been applied successfully to identify disease mechanisms from GWAS or other genomics data for other disorders, including allergic rhinitis and autism spectrum disorder (Ben-David and Shifman 2012; Bunyavanich et al. 2014; Parikshak et al. 2013; Willsey et al. 2013). Admittedly, these studies benefited from having available gene expression data obtained under disease-specific conditions (Bunyavanich et al. 2014) or the use of causal genetic variants with large effect sizes (Ben-David and Shifman 2012; Parikshak et al. 2013; Willsey et al. 2013). For migraine, no gene expression data from disease-conditions are available. Few gene expression profiling studies have been carried out for migraine, i.e., in whole blood of episodic and chronic migraine patients (Hershey et al. 2004) and menstrual migraine patients (Hershey et al. 2012), in immortalised cell lines of migraine with aura patients (Nagata et al. 2009), and in brain material of transgenic KI FHM1 mice (de Vries et al. 2014), but no overlapping deregulated genes or pathways have been identified. Nor is there a large set of causal genes, except for three genes (*CACNA1A, ATP1A2* and *SCN1A*) (De Fusco et al. 2003; Dichgans et al. 2005; Ophoff et al. 1996) that have been identified for FHM, that can guide gene identification efforts in the common forms of migraine. Therefore, we focused our analyses on gene expression data from the normal human brain.

Here we used two complementary methods to connect gene expression data from adult human brain, the most relevant tissue for migraine, with GWAS data to identify migraine-related pathways. To this end, spatially mapped gene expression data of the adult human brain, obtained from the Allen Human Brain Atlas (Hawrylycz et al. 2012), was used to calculate brain-specific co-expression levels between genes. We used GWAS data, available through the International Headache Genetics Consortium, of 23,285 migraine cases and 95,425 population-matched controls (Anttila et al. 2013) to calculate gene-based associations with migraine. This enabled the inclusion of below-threshold association signals that did not reach genome-wide significance (*P* value <5 × 10^−8^) due to lack of power (Gibson 2012; Mooney et al. 2014). For our first method, we grouped all genes into co-expression modules and studied the enrichment of genes with nominally significant gene-based associations with migraine in the different modules. For our second method, we constructed local co-expression networks around ‘high-confidence genes’ (i.e., those genes with gene-based *P* values that survived multiple testing correction) that we combined into a local migraine-related co-expression gene network. By studying the modules enriched for migraine-associated genes (method 1) and the local migraine-related co-expression gene networks (method 2), we identified multiple brain regions, cell types and pathways overlapping between the two methods that are possibly involved in migraine pathophysiology.

## Results

### Spatial co-expression network of the adult human brain

**Fig. 1:**
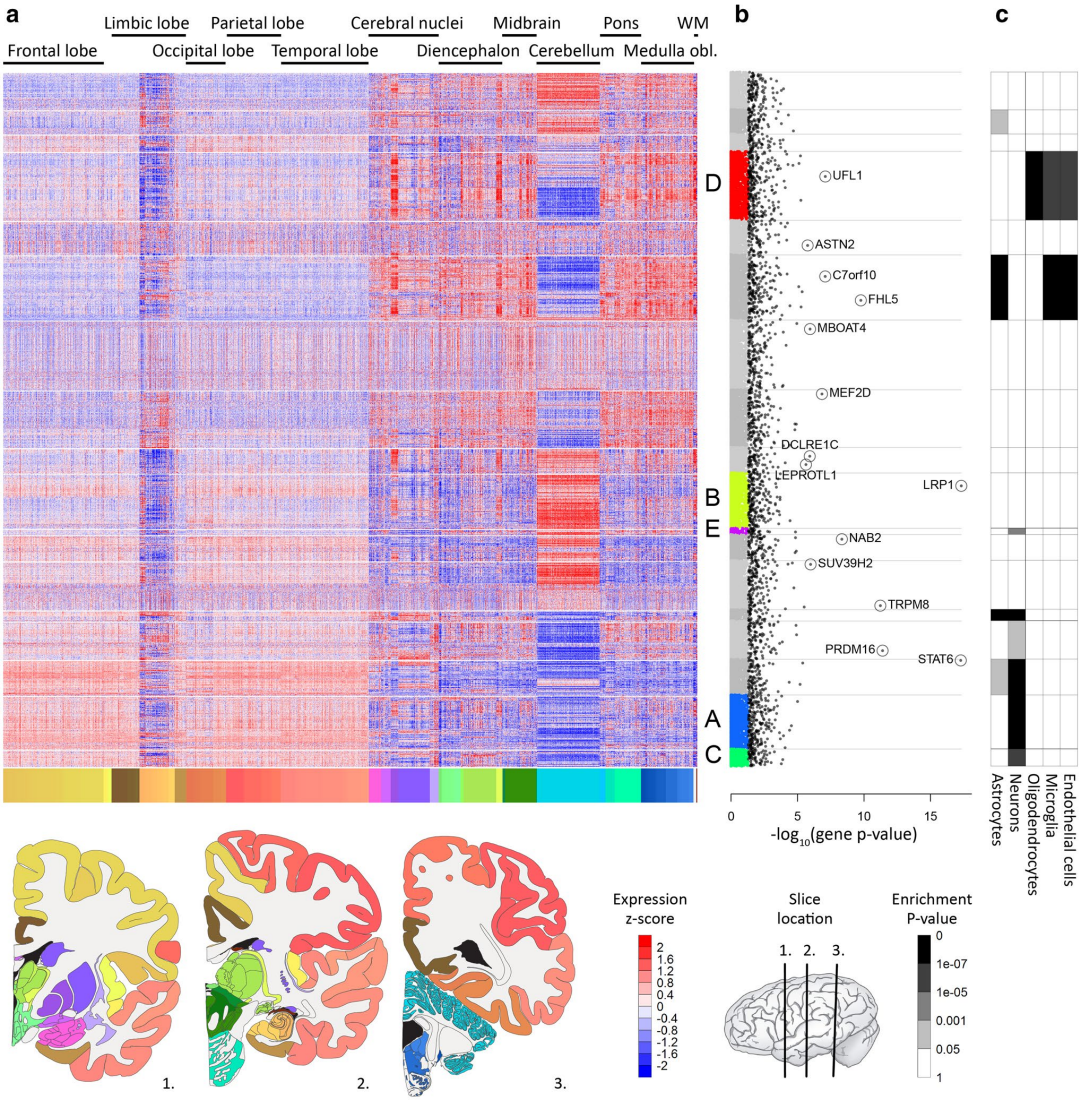
Gene expression patterns and cell type enrichments of the 18 modules in the spatial co-expression network. **a** Heat map of the clustered gene expression data, with the 3702 concatenated human brain samples in columns and the 19,972 genes in rows, ordered according to their clustering. The brain samples are ordered based on their location in the brain, which is noted above the heat map and illustrated with the *colour coding* from the Allen Brain Institute below the heat map. The *colour coding* is also illustrated in the three coronal brain sections below the heat map (for brain region names in the coronal sections, see Figure S3). Low expression is shown as *blue*, high expression is shown as *red*. The genes are clustered into 18 modules, here separated by *white rows*. **b** Log-transformed gene-based *P* values for the association with migraine are shown for all genes with: (1) genes with *P* values below 0.05 in the *colour* corresponding to modules A–E or in *grey* for the other modules; (2) migraine candidate genes in *black*; and (3) high-confidence genes *circled* and *named*. Gene modules* A*–*E* are the five modules enriched for candidate genes. **c** the table shows the enrichment of cell type-specific genes in the 18 modules from *white* (*P* value >0.05) to *black* (*P* value <10^−7^)

To identify brain regions and pathways involved in migraine pathophysiology, we performed co-expression network analysis using spatial gene expression information of the Allen Human Brain Atlas (Hawrylycz et al. 2012). We focused on the adult human brain transcriptome, since migraine is a brain-related disorder that affects mostly the adult population. Microarray data were available from six healthy adult human brains; five males and one female, aged 24–57 with a mean age of 42 years, each dissected into 363–946 samples (3702 in total) from well-defined brain regions. We used the gene expression data of 29,374 microarray probes that could be mapped unambiguously to 19,972 genes. Gene co-expression levels were calculated separately for each brain (across the samples), and subsequently averaged (per gene) to obtain a single spatial co-expression network not affected by individual brain differences (see Materials and methods). Note that these levels, therefore, reflect brain-wide spatial co-expression. Differences in expression values between the female brain and five male brains were not more pronounced than the differences between any of the male brains and all other brains (see Supplementary Materials and methods; Figure S1), justifying the unbalanced gender composition of the Allen Brain Atlas for our analyses. In fact a recent publication by Hawrylycz et al. (2015) showed that functionally relevant genes seem to have a stable expression across the six donors. Using hierarchical clustering analysis, we identified 18 modules in the spatial brain-wide co-expression network, with module sizes varying from 179 to 2007 genes (Fig. 1). Each module thus contains genes that have similar expression patterns across the different brain samples. Clustering the gene expression data can be done in various ways (see Supplementary Materials and methods). The final clustering tree showed strongest enrichment for migraine genes. Modules enriched for migraine genes are further investigated for these spatial patterns across brain regions and for functional enrichments of the migraine genes.

### Genes associated with migraine

We used summary statistics data from the GWAS meta-analysis for migraine (Anttila et al. 2013) performed by the International Headache Genetics Consortium to calculate gene-based *P* values for the association with migraine. The 2116 genes with nominal gene-based *P* values below 0.05 were considered to have a potential link to migraine and are therefore referred to as migraine ‘candidate genes’. The 14 genome-wide significant genes, with multiple testing corrected gene-based *P* values below 0.05, are referred to as ‘high-confidence genes’. The high-confidence genes contained 10 genes located at or near the genome-wide significant GWAS loci: *ASTN2*, *C7orf10*, *FHL5*, *MEF2D*, *TRPM8*, *LRP1*, *STAT6*, *NAB2*, *PRDM16* and *UFL1* (Anttila et al. 2013). *LRP1*, *STAT6* and *NAB2* at chromosome 12q13 share the same genome-wide significant SNP, and the top SNPs for *FHL5* and *UFL1* at chromosome 6q16 are in strong linkage disequilibrium (LD). The remaining high-confidence genes *LEPROTL1, DCLRE1C, SUV39H2*, and *MBOAT4* are located near SNPs that did not reach the level of genome-wide significance in the migraine GWAS, and gain from a reduced multiple testing burden in our gene-based analysis compared to a SNP-based analysis. GWAS hits *MTDH, PHACTR1, TGFBR2, MMP16, TSPAN2* and *AJAP1* did not reach a multiple testing corrected gene-based *P* value below 0.05, possibly due to a larger distance between the GWAS locus and the gene, and were therefore not designated as high-confidence genes.

### Migraine-associated loci converge into five co-expression modules

We performed an enrichment analysis of the 2116 migraine candidate genes in the 18 co-expression modules to identify the modules that have the strongest link with migraine. Five modules labelled A–E showed enrichment of candidate genes in a Fisher exact test (*P* < 0.05) (Fig. 1; Table S1). To verify that the identified enrichments were not the result of bias in the Fisher exact test introduced by LD between SNPs in the GWAS data and by SNPs assigned to multiple genes, we performed a second, LD-corrected Fisher exact test. These results confirm the association of modules A–E with migraine (Table S1).

**Fig. 2:**
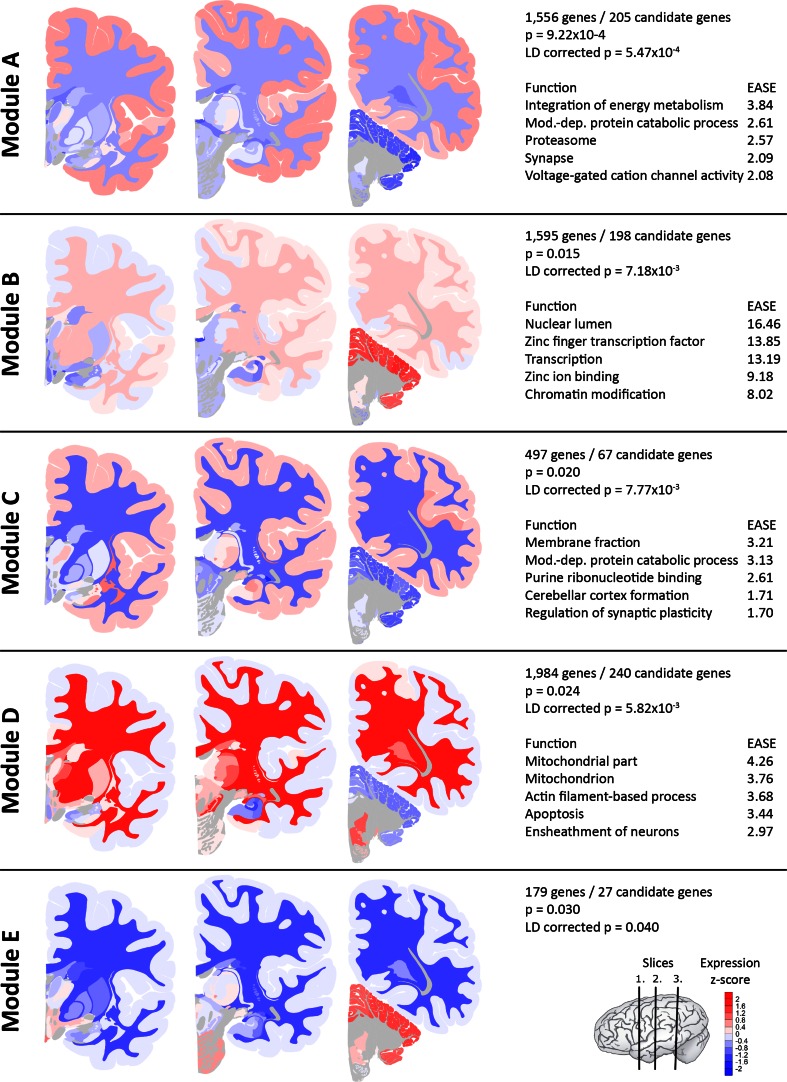
Gene expression maps for modules* A*–*E* associated with migraine. Average gene expression levels are shown for each module from *blue* (low) to *red* (high) in the different brain regions represented in the three coronal brain sections (for brain region names in the coronal sections, see Figure S3). Regions that lack gene expression information are depicted in *grey*. The lists on the right show: (1) the numbers of genes and migraine candidate genes; (2) the *P* values for the enrichment of migraine candidate genes; and (3) the top 5 enriched functions in each module, as identified using the Functional Annotation Clustering tool in DAVID, with their corresponding EASE score. The EASE score is the geometric mean of the Benjamini-corrected negative log (base 10) *P* values of its pathways and GO terms, so a score below 1.3 corresponds to a Benjamini-corrected *P* value below 0.05. Module E has no significant functional enrichments

Module A showed the highest enrichment of migraine candidate genes (enrichment *P* = 9.44 × 10^−4^, LD-corrected enrichment *P* = 5.47 × 10^−4^) and contains 1556 genes with high expression in cerebral cortex, very low expression in cerebellum, and low expression in hippocampal formation and subcortical cerebrum (Fig. 2). Module B (enrichment *P* = 0.015, LD-corrected enrichment *P* = 7.18 × 10^−3^) consists of 1595 genes with high expression in cerebellum, low expression in subcortical regions and an intermediate expression in cerebral cortex (Fig. 2). Module C (enrichment *P* = 0.02, LD-corrected enrichment *P* = 7.77 × 10^−3^) contains only 497 genes. Genes from module C have an expression pattern similar to that of module A with higher expression in hippocampal formation and claustrum (Fig. 2). Module D (enrichment *P* = 0.024, LD-corrected enrichment *P* = 5.82 × 10^−3^) is the largest module with 1984 genes that are preferentially expressed in subcortical regions and the white matter, with low expression in cerebellar and cerebral cortex (Fig. 2). Module E (enrichment *P* = 0.03, LD-corrected enrichment *P* = 0.04) contains only 179 genes with high expression in cerebellar cortex, pons and hypothalamus (Fig. 2).

### Migraine-associated modules show enrichment of functions involved in neurotransmission, mitochondria, gene expression regulation and oligodendrocytes

Next, we performed a functional enrichment analysis of modules A–E to identify gene functions associated with migraine pathophysiology (Fig. 2; Tables S2–S5). We studied pathways from KEGG, Reactome and PANTHER, and gene ontology (GO) terms from PANTHER and the GO FAT database using the Functional Annotation Clustering tool in DAVID. GO term and pathway groups were considered significant when the Benjamini-corrected *P* value was below 0.05 (reflected in an EASE score of 1.3 or higher). Functions enriched in module A included energy metabolism, protein catabolism and synaptic functions (Table S2). Genes in module B showed enrichment of multiple functions, all involved in gene expression regulation (Table S3). Module C contains a large set of genes involved in purine nucleotide binding, and also showed enrichment for several brain developmental and synaptic functions (Table S4). Genes in module D showed highest enrichment of functions involving energy supply, apoptosis and myelination (Table S5). Module E did not show any significant functional enrichments. Most enriched functions are module-specific; of modules A–D only module C shares most of its enriched functions with other modules (A, L and P) (Figure S2).

### Enrichment of oligodendrocytic and neuronal genes in migraine-associated modules

Expression patterns in the brain are co-determined by cell type composition (Grange et al. 2014; Hawrylycz et al. 2012). Consequently, we expected to find enrichment of cell type-specific genes in the co-expression modules (Fig. 1). Notably, modules A and C showed significant enrichment of genes specifically expressed in neurons (119 genes, *P* = 8.00 × 10^−15^; 40 genes *P* = 3.12 × 10^−6^, respectively), which is in line with the preferential expression in cerebral cortex of genes in these modules and the enrichment for synaptic functions. Module D is significantly enriched for oligodendrocyte-specific genes (103 genes, *P* = 1.37 × 10^−55^), and also showed enrichment for genes specifically expressed in microglia and endothelial cells. This finding seems well in line with the observed high expression in white matter of genes in this module and the enrichment of several functions related to myelination. Module E is enriched for neuron-specific genes (18 genes, *P* = 1.09 × 10^−4^). Module B did not show enrichment of cell type-specific genes.

### Confirmation of the association of modules A–D with migraine using a local seed network

**Fig. 3:**
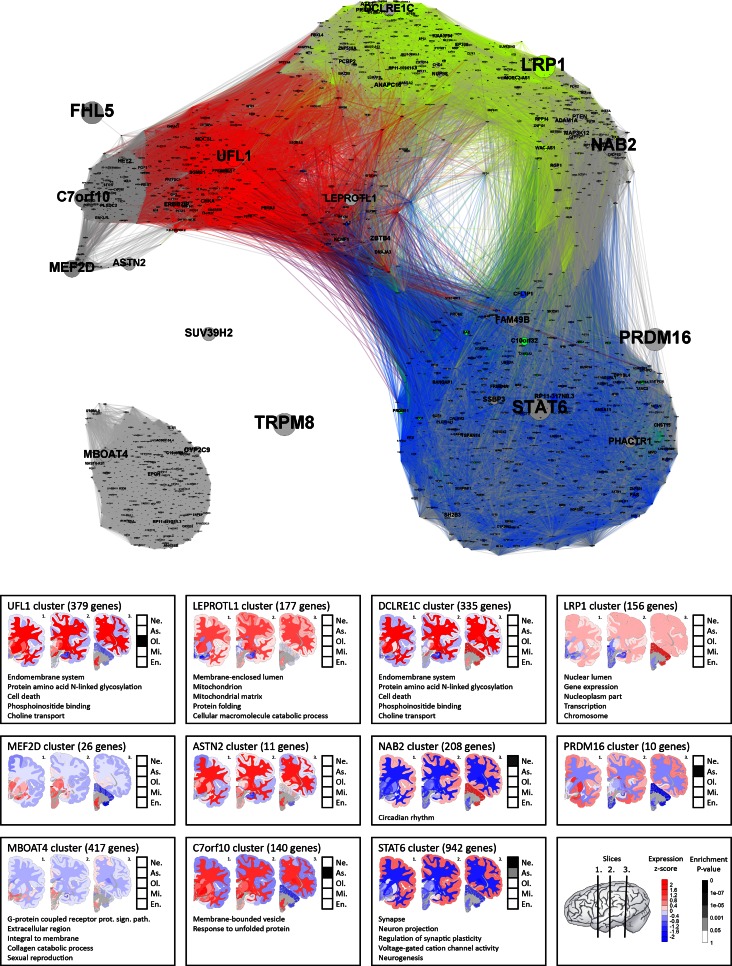
Gene co-expression network seeded on the 14 high-confidence genes. **a** The network consists of the high-confidence genes and their co-expression partners that are connected if they have a co-expression value >0.6. Each gene is shown as a *circle* and named with its gene name, with the size of both corresponding to its gene-based *P* value (larger size corresponding to a lower *P* value). The *colours* of the *circles* correspond to those of modules* A*–*E* in Fig. 1: *blue* for module A, *yellow* for module B, *green* for module C, *red* for module D, *purple* for module E and *grey* for all other modules. The *edge colours* are matched to (a mixture of) the colours of the connecting genes. **b** For each high-confidence gene and its co-expressing partners are shown: (1) the number of genes in the local co-expression network around the high-confidence gene; (2) the average brain gene expression level from *blue* (low expression) to *red* (high expression) mapped in the three coronal brain sections (for brain region names in the coronal sections, see Figure S3); (3) the enrichment of cell type-specific genes in the table from *white* (*P* value >0.05) to *black* (*P* value <10^−7^); and (4) the top five enriched gene functions. Not shown are boxes for high-confidence genes *TRPM8,* *SUV39H2* and *FHL5* because these genes have no or only few co-expressed genes. *Ne*. Neuron, *As*. astrocyte, *Ol*. Oligodendrocyte, *Mi*. microglia, *En*. endothelial cell

The association of modules A–E with migraine may be the result of low migraine association signals, and may therefore not have a direct link to the genome-wide significant GWAS loci, as only module B (*LRP1*) and module D (*UFL1*) contain a high-confidence gene (Fig. 1). To leverage the information in the high-confidence genes, we used them as seeds for a local co-expression network. The local co-expression network therefore contains only the high-confidence genes and their co-expression partners (Fig. 3).

The most highly connected high-confidence gene is *STAT6*, which has strong co-expression with genes from module A (connections marked in blue in Fig. 3) and two genes from module C (connections marked in green), but is not part of either of these modules. Genes *DCLRE1C* and *LRP1* lie in a sub-network containing genes from module B (connections marked in yellow). *LEPROTL1* and *UFL1* are directly connected to genes from module D (marked in red). *SUV39H2* and *TRPM8* have no strongly co-expressed genes in the Allen Human Brain Atlas and remain unconnected. *MBOAT4* lies in a disconnected sub-network. The remaining 6 high-confidence genes are indirectly connected to the genes of modules A–D. The smallest module of interest, module E, has no genes in the local seed network.

### Local seed network shows enrichment of functions and cell types similar to modules A–D

We performed a functional enrichment analysis in the local seed network, thereby focussing on each high-confidence gene and its co-expressing partners (Fig. 3; Table S6). Briefly, a local network for each high-confidence gene was constructed by connecting it to genes with which it has a spatial gene co-expression larger than 0.6. The network around *STAT6,C7orf10* and *MBOAT4* showed enrichment of functions involved in the synapse and signal transduction. The network around *LEPROTL1* showed enrichment of mitochondrial genes. Functions involved in gene expression regulation were found in the networks around *DCLRE1C, LRP1* and *UFL1*. Other enriched functions were “circadian rhythm” (*NAB2* network), “apoptosis” (*UFL1* network), and “protein catabolism” (*LEPROTL1* network).

Finally, we investigated the enrichment of brain cell type-specific genes in the local seed network (Fig. 3; Table S7). The co-expression network around *STAT6*, that shares many genes with module A, is highly enriched for neuron-specific genes (*P* = 4.37 × 10^−32^), as is the network around *NAB2* (*P* = 2.50 × 10^−4^). The sub-network connected to *UFL1*, overlapping with module D, contains many oligodendrocyte-specific genes (*P* = 1.26 × 10^−8^). The sub-networks connected to *PRDM16* and to *C7orf10* are enriched for astrocyte-specific genes (*P* = 3.82 × 10^−7^ and 4.34 × 10^−10^, respectively).

## Discussion

**Fig. 4:**
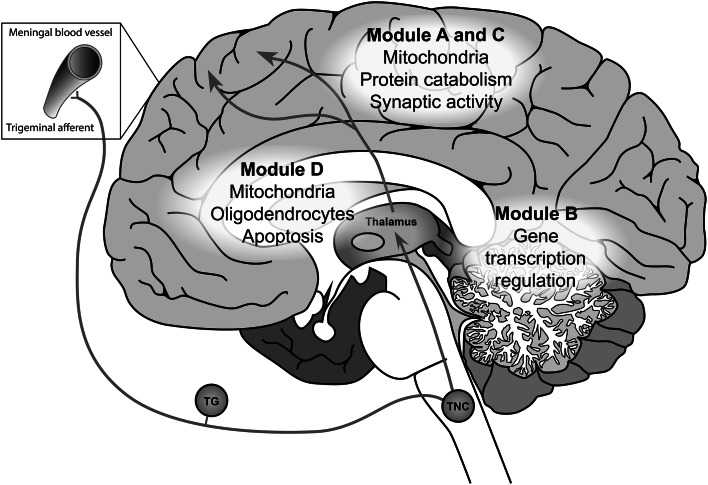
Schematic overview of the migraine-associated modules and the trigeminovascular pathway involved in migraine headache. The migraine-associated modules* A*–*D*, which also overlap with the local migraine-related co-expression gene network, point to three distinct locations in the brain: the cortex (modules A, B and C), the cerebellum (module B) and the white matter and subcortical regions including the thalamus (module D), and multiple gene functions or cell types. Several brain regions overlap between the migraine-associated modules and the trigeminovascular system that is thought to generate the migraine headache. This system consists of trigeminal afferents that innervate the blood vessels in the meninges, whose signals are transmitted through the trigeminal ganglion (TG), the trigeminal nucleus caudalis (TNC), and the thalamus to the cortex where they can produce the sensation of pain

We performed a gene-based analysis of migraine GWAS data from a large meta-analysis of in total 23,285 migraine cases and 95,425 population-matched controls available through the International Headache Genetics Consortium (Anttila et al. 2013) aimed at identifying brain regions, cell types and pathways involved in migraine pathophysiology. To this end, we used detailed spatial brain gene expression data from 3702 samples of six normal adult human brains from the Allen Human Brain Atlas to group genes into co-expression modules. We identified five modules enriched for migraine-associated genes that show involvement in cortical neurotransmission, protein catabolism and energy supply (Modules A and C); in gene transcription regulation in cortex and cerebellum (Module B); and in myelination and energy supply in subcortical areas (Module D) (Fig. 4).

The lack of causal variants with large effect sizes for common migraine may explain, at least partly, the low enrichments of candidate genes in the co-expression modules. The conversion of the migraine GWAS data to the gene-based *P* values may have caused inaccuracies as we may have associated SNPs to genes just because they are nearby these genes, although they may not have a functional effect on them; and, similarly, we may not have associated SNPs to genes simply because we considered them too far away to be functionally involved. To reduce these limitations we chose a 15-kb boundary around the genes, as it was shown that most SNPs that affect gene expression are located within this boundary (Pickrell et al. 2010). However, currently, no methods are available to calculate gene-based *P* values that can fully surmount these limitations. To increase the reliability of our results, we used the largest migraine GWAS dataset currently available (Anttila et al. 2013). Furthermore, we used a second method to confirm the link between migraine and the brain regions and gene functions identified by building a migraine-related co-expression gene network around the high-confidence migraine genes. Although the enrichment of migraine-associated genes in the modules cannot proof that these brain regions, cells and pathways are dysfunctional in migraine patients, it can provide genetic evidence for processes already implicated in migraine, and may indicate new areas of interest for migraine research.

Two modules enriched for migraine-associated genes contained genes highly expressed in cortex that are largely involved in neurotransmission and that are highly enriched for neuron-specific genes (Modules A and C). Furthermore, module A contains many components of the glutamatergic system (*GLS, GRIK3, GRIN2A* and *GRM7*). The cell type enrichments in the modules were based on gene expression data from isolated mouse brain cells (Zhang et al. 2014). Similar data from mouse studies have been used previously for characterisation of human brain co-expression modules (Hawrylycz et al. 2015). These results confirm the link between cortical neurotransmission and migraine that had previously been identified in genetic studies in FHM (Ferrari et al. 2015). Several genes (*MTDH*, *LRP1*, *MEF2D*) identified by GWAS hits for common migraine could also be linked to glutamate signalling (Tolner et al. 2015), although these genes are not part of modules A or C.

The enrichment of genes involved in mitochondria in modules A and D form the first *genetic* link between mitochondrial function and common migraine. As neurotransmission requires a large amount of energy, it is not surprising that mitochondrial deficiencies have been implicated in a wide range of neurological disorders, including migraine (Sparaco et al. 2006). In migraine patients, magnetic resonance spectroscopy studies have consistently identified a depletion of brain high-energy phosphates, indicative of a disturbed energy metabolism (Reyngoudt et al. 2012). Impaired mitochondrial activity has also been found in muscle and platelets of migraine patients (Reyngoudt et al. 2012; Sangiorgi et al. 1994). Also the efficacy of riboflavin and coenzyme Q10, two enhancers of mitochondrial function, in migraine prophylaxis in two small clinical trials points towards a possible causal role for mitochondria in migraine (Sandor et al. 2005; Schoenen et al. 1998).

Module B shows high expression in cerebellum and medium expression in cortex, and is highly enriched for genes involved in aspects of gene expression regulation (i.e., transcription factors, chromatin remodellers, RNA processing). Migraine pathophysiology has already been associated with actions of a specific set of transcription factors, i.e., female hormone receptors and receptors for the stress hormone cortisol (MacGregor 2004; Sauro and Becker 2009). Although the stress hormone receptor gene *NR3C1* is a member of module B, the other stress hormone receptor gene *NR3C2* and the female hormone receptor genes *ESR1*, *ESR2*, *RXFP1*, *RXFP2* and *PGR* are members of modules F, N, M, P, R and H, respectively. These transcription factors can thus not explain the association of module B with migraine. As to the high expression in cerebellum, there are several lines of evidence that indicate a role for the cerebellum in migraine. (Subclinical) cerebellar abnormalities have been recognised in migraine patients, including lack of fine coordination (Sandor et al. 2001) and vestibulocerebellar problems (Harno et al. 2003). Furthermore, studies using magnetic resonance imaging (MRI) identified cerebellar infarcts (Kruit et al. 2004) and microstructural cerebellar abnormalities (Granziera et al. 2013) in migraine patients. Cerebellar mechanisms causative of migraine are not known, but may possibly include signalling cascades that regulate gene expression as identified in module B.

Module D contains genes highly expressed in several subcortical brain regions and in the white matter and is enriched for gene functions involving myelin formation and genes specifically expressed in oligodendrocytes. Oligodendrocytes play key roles in the formation of axons and neuronal connections (Debanne et al. 2011), and can also actively communicate with neurons to regulate their activity (Butt et al. 2014; Fields 2008; Stys 2011). The genes from module D are expressed in multiple brain regions that are implicated in the processing of migraine pain signalling: the trigeminovascular pathway (Noseda and Burstein 2013). This pathway transmits nociceptive signals from meninges to thalamus and higher brain areas via several brainstem nuclei, including the trigeminal nucleus caudalis (TNC), (Fig. 4). A recent study identified disrupted myelin sheets in the trigeminal nerve of migraine patients (Guyuron et al. 2014), providing first evidence for disturbed oligodendrocyte functioning in the trigeminovascular pathway. Furthermore, a high-field MRI study identified thalamic microstructural abnormalities in migraine patients that could indicate an increase of myelin (Granziera et al. 2014).

In summary, we performed a gene-based analysis of the migraine GWAS data, using detailed spatial gene expression data to define gene modules with similar expression patterns in the normal human brain. Our results showed enrichment of migraine-associated genes in modules involved in cortical neurotransmission, mitochondrial and oligodendrocyte function that provide further evidence that these mechanisms play a causal role in migraine and deserve to be investigated in more detail by (functional) studies in patients and experimental animal models.

## Materials and methods

### GWAS dataset

Summary statistics of migraine GWAS data from 23,285 cases and 95,425 controls from the meta-analysis (Anttila et al. 2013) available through the International Genetics Headache Consortium were used for this study. The quality control of the genotype data was described previously (Anttila et al. 2013). Autosomal SNPs were imputed against the HapMap CEU population (release 21–24 depending on the cohort). To convert the genomic coordinates of the SNPs from human reference genome build 36 to build 37, we used CrossMap (http://crossmap.sourceforge.net/) (Zhao et al. 2014). A total of 1,853,579 SNPs with high quality GWAS data and converted to build 37 were used in the calculation of gene-based *P* values.

### Gene-based *P* values

Gene-based *P* values were calculated from GWAS data using the gene-based test GATES (Li et al. 2011) implemented in the whole-genome analysis platform Fast ASsociation Test (FAST) (Chanda et al. 2013). GATES is a Simes test extension that integrates SNP *P* values into a gene-based test statistic, based on SNP positions and LD information [1000 Genomes data (Phase 1)] by taking the top SNP per gene and correcting its *P* value for the effective number of independent tests. Gene location information based on the GRCh37.p13 build reference sequence was obtained from Biomart (version 75: Feb 2014 archive site). A flanking region of 15 kb up- and downstream of the gene was used to include SNPs located in regulatory regions. The size of the flanking region was based on the identification that most SNPs that influence the expression of a gene are located within 15 kb of the gene (Pickrell et al. 2010). Genes with a gene-based *P* < 0.05 were considered migraine ‘candidate genes’; genes with a Bonferroni corrected *P* < 0.05 were considered ‘high-confidence genes’.

### Spatial gene expression

Spatial gene expression data from six healthy adult human brains was obtained from the Allen Human Brain Atlas (http://human.brain-map.org/) (Hawrylycz et al. 2012). For each brain, RNA had been extracted from 363 to 946 different brain samples and measured on custom Agilent microarrays containing the 4 × 44 K Agilent Whole Human Genome probes as well as an additional 16,000 custom probes. The expression data was matched to the GATES output based on Biomart associations of 4 × 44 K Whole Genome microarray probe IDs with genes. If a probe was matched to multiple genes, it was excluded from the analysis. If multiple probe IDs were associated with the same gene, average expression levels were calculated for that gene. The spatial expression of a gene for a particular brain is thus described by the expression levels of that gene across all samples in that brain. Since the number of brain samples differs per brain, the spatial gene expression vector of a gene differs in length between brains.

### Spatial gene co-expression and hierarchical clustering

Spatial co-expressions between genes were first calculated for each brain separately. For this, robust bi-weight mid-correlations were calculated across all brain samples for each of the six donors separately (Langfelder and Horvath 2012). Subsequently, these correlations were averaged across the donors to obtain co-expression values that only reflect spatial expression patterns and ignore between-brain differences. We then performed hierarchical clustering to obtain modules of spatially co-expressed genes. The linkage and distance measures, and the threshold at which the tree is cut, were chosen to maximise the enrichment of migraine candidate genes (see Supplementary Materials and methods for different combinations of linkage and distance measures). We chose for this independent evaluation over traditional cluster evaluation measures [like WGCNA (Zhang and Horvath 2005)] as we are interested in finding modules (clusters) that are related to migraine genes. Eventually, clustering was done with complete linkage, with one minus the bi-weight mid-correlation as a distance measure, and the tree was cut into 18 clusters.

### Enrichment of candidate genes in the modules

Enrichment of migraine-associated genes within a module was determined using a Fisher exact test that calculated whether the number of migraine candidate genes in a module is higher than expected based on the total number of genes and migraine candidate genes. Neighbouring genes on the genome might have similar expression patterns due to local regulatory DNA elements, as well as similar gene-based *P* values due to LD between their top SNPs or overlapping flanking regions. Therefore, we performed a second LD-corrected Fisher exact test in which we included only the number of independent genes in the calculation. As a measure for the number of independent genes in a gene set, we took the top SNP of each gene and used the Genetic type I Error Calculator (GEC) (Li et al. 2012) to calculate the effective number of independent SNPs based on LD information from the HapMap project release 23. In this way, the LD-corrected Fisher exact test had as input the corrected estimates for the number of independent genes with gene-based *P* values below and above 0.05, both in the cluster of interest and in the full set of genes. See Supplementary Materials and methods for additional information on the enrichment analysis.

### Functional annotation

Gene ontology (GO) term and pathway enrichment analysis in the modules was performed with DAVID (version 6.7; http://david.abcc.ncifcrf.gov/). We used the Functional Annotation Clustering tool in DAVID to group significant GO terms and pathways based on co-associated genes to remove redundant terms (Huang et al. 2007). Pathway information from KEGG, Reactome and PANTHER, and GO term information (biological processes, molecular functions and cellular components) from PANTHER, and the FAT subsets of GO terms was used. GO term and pathway groups were considered significant when the EASE score was larger than 1.3 (corresponding to a geometric mean Benjamini-corrected *P* value of the clustered GO terms and pathways below 0.05). Significant groups were named after the most significant term in the group. Comparison of GO term and pathway enrichments between modules was performed in ToppCluster, a multiple gene list feature enrichment analyser (Kaimal et al. 2010). In ToppCluster, we performed GO term (biological processes, molecular functions and cellular components) and pathway enrichment analyses for all modules, which were considered significant when Bonferroni-corrected *P* values were below 0.05. Functional enrichments and overlap in enrichments between modules were visualised in Cytoscape (version 3.2.1).

### Cell type enrichment

For enrichment analysis of cell type-specific genes we made use of cell type-specific genes identified in gene expression data from isolated mouse brain cells (Zhang et al. 2014). We selected the gene expression data from neurons, astrocytes, myelinating oligodendrocytes, microglia, and endothelial cells. Genes were considered cell type-specific if they had more than tenfold higher gene expression [reads per kb per million (RPKM)] levels compared to the mean expression in the other cell types. We obtained 818 neuron-, 380 astrocyte-, 198 oligodendrocyte-, 692 microglia-, and 546 endothelial-specific genes for which human orthologs were present. Enrichment was determined with Fisher exact tests.

### Local modules from seed genes

Local co-expression networks were built from high-confidence genes by adding genes to the network whose co-expression exceeds a threshold [similar to Willsey et al. (2013)]. Genes were only selected if they had co-expression values higher than 0.6 with a high-confidence gene. The threshold was chosen to: (1) maintain only reasonably strong links between genes, especially given the fact that we use robust bi-weight mid-correlations; and (2) have linking genes for most of the seed genes (see Supplementary Materials and methods for information on how the threshold value was selected). Co-expressions were measured as bi-weight mid-correlations, the same co-expression values which were used to determine the genome-wide co-expression modules, and local modules were defined as all genes connected to a single high-confidence gene. If a gene is connected to two high-confidence genes, it is part of the modules of both genes.

## Electronic supplementary material

The Supplementary Information contains the Supplementary Materials and methods, the Supplementary Tables S1-S8, and Supplementary Figures S1-S4. (PDF 517 kb)
