# Supplementary material for: Gene co-expression analysis identifies brain regions and cell types involved in migraine pathophysiology: a GWAS-based study using the Allen Human Brain Atlas
Source: Hum Genet. 2016 Feb 22;135:425–39. doi: 10.1007/s00439-016-1638-x (PMC4796339; doi:10.1007/s00439-016-1638-x)
Supplement: Supplementary file 1 — The Supplementary Information contains the Supplementary Materials and methods, the Supplementary Tables S1-S8, and Supplementary Figures S1-S4. (PDF 517 kb) [file 439_2016_1638_MOESM1_ESM.pdf]

Supplemental Information

Gene co-expression analysis identifies brain regions and cell types involved in migraine pathophysiology: a GWAS-based study using the Allen Human Brain Atlas

Else Eising*, Sjoerd M.H. Huisman*, Ahmed Mahfouz, Lisanne S. Vijfhuizen, on behalf of the International Headache Genetics Consortium: Verneri Anttila, Bendik S. Winsvold, Tobias Kurth, M. Arfan Ikram, Tobias Freilinger, Jaakko Kaprio, Dorret I. Boomsma, Cornelia M. van Duijn, Marjo-Riitta R. Järvelin, John-Anker Zwart, Lydia Quaye, David P. Strachan, Christian Kubisch, Martin Dichgans, George Davey-Smith, Kari Stefansson, Aarno Palotie; Daniel I. Chasman, Michel D. Ferrari, Gisela M. Terwindt, Boukje de Vries, Dale R. Nyholt, Boudewijn P.F. Lelieveldt#, Arn M.J.M. van den Maagdenberg#, Marcel J.T. Reinders#

* These authors contributed equally to this work.
# These authors jointly directed this work.

Supplemental Material and Methods

Hierarchical clustering

To identify modules of genes with high relevance to biological processes in the brain, we clustered all genes based on their co-expression in the normal adult human brain (using data from the Allen Human Brain Atlas www.brain-map.org). We performed hierarchical clustering, for which choices need to be made on a number of elements, such as distance measure and linkage method.

The first element is the conversion of probe expression values to gene expression values. This was performed in one of two ways, we either took 'the mean expression of all probes belonging to a gene' or 'the expression values of the probe with the highest variance', measured as the median of the six within-donor expression variances. The assumption would be that probes with a higher variance are more likely to represent a strong signal.

The second element is the similarity measure to define co-expression. This was also performed in one of two ways, either using 'Pearson correlation' or 'bi-weight mid-correlation'. In bi-weight mid-correlation gene expression values that are much higher or lower than the median value are given less

weight in the correlation calculation, which makes the method more robust to extreme values and outliers (Langfelder and Horvath 2008).

To reduce the influence of non-informative genes on the gene clustering, we also considered filtering out genes. We either performed ‘no filtering’, ‘filtering based on expression variance’, or ‘filtering on co-expression’. For the variance filtering, the expression variance was calculated within each of the six donors. Genes with a median variance within the 10th lower percentile were filtered out. For the co-expression filtering, genes were removed when they had no similarity and/or co-expression values higher than 0.7 to any of the other genes.

The final element in the clustering is the linkage method (to define distances between clusters of genes). Three methods were tried: ‘average linkage’, ‘complete linkage’, and ‘Ward’s linkage’. In average linkage the average gene-gene distance is used as a distance between clusters, in complete linkage this is the largest gene-gene distance. Ward’s method minimizes within cluster variance (Murtagh and Legendre 2014; R Core Team 2014).

The combinations of these elements gave 36 trees, which were all cut at varying heights, and enrichment of migraine ‘candidate genes’ was determined with a Fisher exact test. The final selected tree, with strongest enrichments, was that with probe to gene conversion using mean expressions, bi-weight mid-correlation as a similarity, no gene filtering, and complete linkage. The final tree was cut into 18 clusters to have a balance between strength of migraine enrichments and sizes of the clusters.

Enrichment analysis

The co-expression modules were tested for enrichment of migraine ‘candidate genes’, i.e. genes with unadjusted *P*-values below 0.05 for the association with migraine. The enrichment analysis was performed with a Fisher Exact test. It takes the number of genes within a module of interest, the number of ‘candidate genes’ and the total number of genes, to give an exact *P*-value for association between the module and the ‘candidate genes’ (assuming random assignment of ‘candidate genes’ to modules). The one-sided test gives a low *P*-value if genes in the module are more often ‘candidate genes’ than to be expected by chance.

Genes that are close together on the genome are more likely to be co-expressed, and they may share SNPs of interest due to linkage disequilibrium (LD). To correct for LD we calculated the effective independent number of genes using the Genetic type I Error Calculator (GEC) (Li et al. 2012). These numbers were then used in the Fisher exact tests instead of the real numbers of genes.

Differences between donor brains

Migraine is more prevalent in females, which raises the question how much the co-expression patterns in the brain are influenced by the sex of the donor brains. To investigate this, we analysed the proportion of genes that are differentially expressed between the female brain and the male brains. To do so we calculated, for each gene, the average expression in each of the 105 brain regions that were sampled in all six donors (see Table S8), resulting in six expression values for each gene-region combination, one per donor.

We then performed a *t*-test for differential expression for each gene-region combination, testing one brain versus all five other brains. So for the female brain, we compared the expression in the female brain to the expression in the five male brains (Figure S1). If a gene is differentially expressed (*P*-value < 0.05), it has a significantly higher or lower expression in this brain region for this donor than for the other five donors. The proportion of differentially expressed genes per region-donor combination gives an indication of how different expression is in the specific donor (with respect to the remaining donors). The female brain has more than 5% differentially expressed genes in some regions, but this also happens a lot when comparing a male brain to the remaining donors. From that we conclude that the co-expression values are not driven by sex-specific differences.

Co-expression network threshold

The local co-expression module approach requires the specification of a threshold, to determine which genes are co-expressed to the high confidence seed genes. For a very high value of this threshold, many seed genes will not be connected to any other genes, which means we cannot learn from their transcriptional context. For low values of the threshold, seed genes will also be connected to irrelevant genes. To determine a suitable threshold, we analysed the connectivity of each seed gene (i.e. the number of connections it has in the local network). Figure S4 shows the connectivity values for a range of thresholds. The threshold of 0.6 was chosen because the most connected gene (STAT6) has less than 1000 connections at this threshold, and most of the other seed genes remain connected to other genes in the network.

Supplemental Tables

Supplemental Table 1: Enrichment of 'migraine candidate genes' in the co-expression modules

Module	# Genes	# Candidate genes	*P*-value for enrichment	LD-corrected *P*-value for enrichment
A	1,556	205	9.22×10^{-4}	5.47×10^{-4}
B	1,595	198	0.015	7.18×10^{-3}
C	497	67	0.020	7.77×10^{-3}
D	1,984	240	0.024	5.82×10^{-3}
E	179	27	0.030	0.040
F	497	60	0.164	0.122
G	750	87	0.238	0.143
H	1,875	200	0.450	0.220
I	736	78	0.521	0.259
J	1,080	113	0.573	0.463
K	705	70	0.714	0.328
L	326	30	0.826	0.707
M	1,411	138	0.833	0.582
N	1,656	157	0.902	0.762
O	1,101	102	0.941	0.795
P	1,024	94	0.968	0.877
Q	993	86	0.982	0.95
R	2,007	164	1.000	0.998

The enrichment analysis of migraine 'candidate' genes in the 18 co-expression modules, using the Fisher exact test, and the LD-corrected Fisher exact test.

Supplemental Table 2: Functions enriched in module A

Cluster	# Genes	EASE score
Integration of energy metabolism	225	3.84
Modification-dependent protein catabolic process	102	2.61
Proteasome	184	2.57
Synapse	63	2.09
Voltage-gated cation channel activity	112	2.08
Mitochondrial part	68	1.99
Neuron projection	47	1.75
Response to calcium ion	22	1.47
Oxaloacetate metabolic process	7	1.43
Electron transport	53	1.36

The Functional Annotation Clustering tool in DAVID was used to identify functions enriched in Module A (1,556 genes). Pathway information from KEGG, Reactome and PANTHER, and GO term information (biological processes, molecular functions and cellular components) from PANTHER and the FAT subsets of GO terms was used in the analysis. The name of each cluster was based on the most significant pathway or GO term within that cluster. The EASE score is the mean of the Benjamini-corrected negative log (base 10) *P*-values of its pathways and GO terms, so a score of 1.3 corresponds to a Benjamini-corrected geometric mean *P*- value of 0.05.

Supplemental Table 3: Functions enriched in module B

Cluster	# Genes	EASE score
Nuclear lumen	223	16.46
Zinc finger transcription factor	215	13.85
Transcription	423	13.19
Zinc ion binding	388	9.18
Chromatin modification	80	8.02
RNA processing	157	6.79
Chromosome	64	4.75
Non-membrane-bounded organelle	229	4.36
Methyltransferase	35	3.35
Transcription activator activity	92	3.27
DNA metabolic process	92	3.11
Negative regulation of gene expression	133	3.09
RNA biosynthetic process	41	2.42
Ribonucleoprotein complex biogenesis	52	2.10
DNA repair	44	1.95
Androgen receptor binding	18	1.91
Histone modification	36	1.89
Nuclear-transcribed mRNA catabolic process	12	1.87
Macromolecule catabolic process	111	1.82
Chromosome, centromeric region	26	1.77
DNA-directed RNA polymerase	42	1.66
Regulation of gene expression, epigenetic	19	1.36
Regulation of gene expression, epigenetic	17	1.35
Chromatin assembly or disassembly	27	1.34
DNA replication	27	1.33

The Functional Annotation Clustering tool in DAVID was used to identify functions enriched in Module B (1,595 genes). The EASE score is the mean of the Benjamini-corrected negative log (base 10) *P*-values of its pathways and GO terms, so a score of 1.3 corresponds to a Benjamini-corrected geometric mean *P*-value of 0.05.

Supplemental Table 4: Functions enriched in module C

Cluster	# Genes	EASE score
Membrane fraction	45	3.21
Modification-dependent protein catabolic process	47	3.13
Purine ribonucleotide binding	128	2.61
Cerebellar cortex formation	9	1.71
Regulation of synaptic plasticity	12	1.70
Ubiquitin-protein ligase activity	16	1.40

The Functional Annotation Clustering tool in DAVID was used to identify functions enriched in module C (497 genes). The EASE score is the mean of the Benjamini-corrected negative log (base 10) *P*-values of its pathways and GO terms, so a score of 1.3 corresponds to a Benjamini-corrected geometric mean *P*-value of 0.05.

Supplemental Table 5: Functions enriched in module D

Cluster	# Genes	EASE score
Mitochondrial part	92	4.26
Mitochondrion	273	3.76
Actin filament-based process	68	3.68
Apoptosis	100	3.44
Ensheathment of neurons	62	2.97
Oligodendrocyte differentiation	16	2.35
Regulation of collagen biosynthetic process	7	2.23
Protein complex assembly	92	2.22
Phosphoinositide binding	59	2.17
Basolateral plasma membrane	69	2.15
Organellar ribosome	58	2.03
Membrane organization	80	2.02
Basal part of cell	16	1.99
Endoplasmic reticulum membrane	109	1.96
Cytoskeletal protein binding	86	1.95
Lipid biosynthetic process	77	1.93
Identical protein binding	108	1.89
Brush border	19	1.88
Biological adhesion	111	1.85
Lytic vacuole	41	1.81
Rho gtpase binding	18	1.72
Negative regulation of programmed cell death	104	1.72
Tissue remodeling	24	1.65
Respiratory chain	89	1.63
Positive regulation of signal transduction	54	1.54
Membrane lipid biosynthetic process	15	1.53
Organic acid biosynthetic process	25	1.49
Response to nutrient	32	1.42
Iron ion transport	15	1.42
Protein amino acid glycosylation	46	1.39
Fatty acid beta-oxidation	39	1.36
Cell migration	63	1.34
Regulation of cell morphogenesis	84	1.34
Nucleobase, nucleoside, nucleotide and nucleic acid catabolic process	14	1.34
Translation initiation factor activity	32	1.30

The Functional Annotation Clustering tool in DAVID was used to identify functions enriched in module D (1,984 genes). The EASE score is the mean of the Benjamini-corrected negative log (base 10) *P*-values of its pathways and GO terms, so a score of 1.3 corresponds to a Benjamini-corrected geometric mean *P*-value of 0.05.

Supplemental Table 6: Functions enriched in the local seed network

**DCLRE1C**	
Functions	EASE score
Non-membrane-bounded organelle	7.20
Nuclear lumen	6.57
Mrna metabolic process	5.62
Zinc ion binding	4.08
Nucleoside, nucleotide and nucleic acid metabolism	4.01
Response to DNA damage stimulus	2.74
Chromatin organization	2.62
Chromatin organization	2.53
Nuclear body	1.75
Acetyltransferase	1.60
Helicase	1.54
Nucleocytoplasmic transporter activity	1.50

**MBOAT4**	
Functions	EASE score
G-protein coupled receptor protein signaling pathway	8.03
G-protein coupled receptor protein signaling pathway	6.67
Extracellular region	5.24
Integral to membrane	3.41
Collagen catabolic process	2.59
Sexual reproduction	1.75

**NAB2**	
Functions	EASE score
Circadian rhythm	1.42

**C7orf10**	
Functions	EASE score
Membrane-bounded vesicle	1.44
Response to unfolded protein	1.33

**LRP1**	
Functions	EASE score
Nuclear lumen	6.96
Gene expression	2.40
Nucleoplasm part	2.22
Transcription	1.92
Chromosome	1.73

**UFL1**	
Functions	EASE score
Endomembrane system	1.97
Protein amino acid N-linked glycosylation	1.96
Cell death	1.61
Phosphoinositide binding	1.44
Choline transport	1.44
Insoluble fraction	1.39
Actin binding cytoskeletal protein	1.36
Organic acid biosynthetic process	1.35
Lysosome	1.34
Pausing and recovery of Tat-mediated HIV-1 elongation	1.31

**LEPROTL1**	
Functions	EASE score
Membrane-enclosed lumen	5.08
Mitochondrion	4.58
Mitochondrial matrix	4.39
Protein folding	2.01
Cellular macromolecule catabolic process	2.01
Positive regulation of protein ubiquitination	1.54

**STAT6**	
Functions	EASE score
Synapse	8.81
Neuron projection	8.22
Regulation of synaptic plasticity	3.56
Voltage-gated cation channel activity	3.55
Neurogenesis	3.42
Synaptic transmission	3.36
Cytoskeletal protein binding	3.20
Neuron differentiation	2.49
Plasma membrane part	2.08
Exocytosis	2.06
Asymmetric synapse	1.90
Protein modification	1.85
Metal ion transport	1.79
G-protein modulator	1.78
Cerebral cortex gabaergic interneuron differentiation	1.75
Long-term potentiation	1.71
Cerebral cortex gabaergic interneuron differentiation	1.63
Membrane fraction	1.62
Learning or memory	1.57
Synaptic transmission	1.55
Long-term potentiation	1.52
Axon part	1.49
Synaptic vesicle transport	1.44
Neuron maturation	1.36
Regulation of synaptic transmission, glutamatergic	1.35
Amine receptor activity	1.32
Positive regulation of exocytosis	1.32

The Functional Annotation Clustering tool in DAVID was used to identify functions enriched in in the co-expression networks of the 'high-confidence genes'. Pathway information from KEGG, Reactome and PANTHER, and GO term information (biological processes, molecular functions and cellular components) from PANTHER and the FAT subsets of GO terms was used in the analysis. The name

of each cluster is based on the most significant pathway or GO term within that cluster. The EASE score is the mean of the Benjamini-corrected negative log (base 10) *P*-values of its pathways and GO terms, so a score of 1.3 corresponds to a Benjamini-corrected geometric mean *P*-value of 0.05. The local networks around MEF2D, ASTN2 and PRDM16 did not have any functional enrichments. TRPM8 and SUV39H2 do not have co-expressing genes.

Supplemental Table 7: Enrichment of cell type-specific genes in the local seed network

	# Genes	Astrocytes	Neurons	Oligoden-drocytes	Microglia	Endo-thelial cells
**LRP1**	156	1.00	1.00	1.00	0.65	1.00
**STAT6**	942	2.54×10^{-3}	4.37×10^{-32}	0.86	0.57	0.77
**PRDM16**	10	3.82×10^{-7}	1.00	1.00	1.00	1.00
**FHL5**	2	1.00	1.00	1.00	1.00	1.00
**NAB2**	208	0.50	2.50×10^{-4}	0.85	0.94	0.96
**UFL1**	379	0.02	1.00	1.26×10^{-8}	0.91	0.71
**C7orf10**	140	4.34×10^{-10}	0.76	1.00	0.10	0.13
**MEF2D**	26	1.00	1.00	1.00	0.53	0.47
**MBOAT4**	417	1.00	1.00	0.98	1.00	0.57
**DCLRE1C**	335	0.98	1.00	0.81	1.00	0.91
**ASTN2**	11	1.00	1.00	0.10	1.00	1.00
**LEPROTL1**	177	1.00	0.99	1.00	1.00	1.00

Enrichment of cell type-specific genes in the co-expression networks of the ‘high-confidence genes’. TRPM8 and SUV39H2 do not have co-expressing genes. Significant enrichments after Bonferroni correction for multiple testing are marked in bold.

Supplemental Table 8: Acronyms and full names of 105 brain regions used in Supplemental Figure S3

Acronym	Region name
Cl	Claustrum, left
Acb	Nucleus accumbens, left
S	Subiculum, left
CA1	CA1 field, left
CA2	CA2 field, left
CA3	CA3 field, left
CA4	CA4 field, left
DG	Dentate gyrus, left
BCd	Body of caudate nucleus, left
PrG-il	Precentral gyrus, left, inferior lateral aspect of gyrus
SIG	Short insular gyri, left
PrG-prc	Precentral gyrus, left, bank of the precentral sulcus
SFG-l	Superior frontal gyrus, left, lateral bank of gyrus
MFG-s	Middle frontal gyrus, left, superior bank of gyrus
MFG-i	Middle frontal gyrus, left, inferior bank of gyrus
SFG-m	Superior frontal gyrus, left, medial bank of gyrus
MOrG	Medial orbital gyrus, left
LOrG	Lateral orbital gyrus, left
CgGf-s	Cingulate gyrus, frontal part, left, superior bank of gyrus

HCd	Head of caudate nucleus, left
Pu	Putamen, left
Cu	Cuneate nucleus, left
IO	Inferior olivary complex, left
GiRt	Gigantocellular group, left
RaM	Raphe nuclei of medulla
Sp5	Spinal trigeminal nucleus, left
8Ve	Vestibular nuclei, left
LIG	Long insular gyri, left
DTA	Anterior group of nuclei, left
Dt	Dentate nucleus, left
He-VI	VI, left, lateral hemisphere
He-Crus I	Crus I, left, lateral hemisphere
LMRt	Lateral medullary reticular group, left
Arc	Arcuate nucleus of medulla, left
He-Crus II	Crus II, left, lateral hemisphere
FuG-its	Fusiform gyrus, left, bank of the its
PV-V	V, left, paravermis
PV-VI	VI, left, paravermis
PV-Crus I	Crus I, left, paravermis
PV-VIIB	VIIB, left, paravermis
He-VIIB	VIIB, left, lateral hemisphere
PoG-sl	Postcentral gyrus, left, superior lateral aspect of gyrus
CgGp-s	Cingulate gyrus, parietal part, left, superior bank of gyrus
MTG-i	Middle temporal gyrus, left, inferior bank of gyrus
OTG-s	Occipito-temporal gyrus, left, superior bank of gyrus
OTG-i	Occipito-temporal gyrus, left, inferior bank of gyrus
MTG-s	Middle temporal gyrus, left, superior bank of gyrus
LiG-pest	Lingual gyrus, left, peristriate
LiG-str	Lingual gyrus, left, striate
PrG-sl	Precentral gyrus, left, superior lateral aspect of gyrus
PoG-cs	Postcentral gyrus, left, bank of the central sulcus
ATZ	Amygdalohippocampal transition zone, left
STG-l	Superior temporal gyrus, left, lateral bank of gyrus
STG-i	Superior temporal gyrus, left, inferior bank of gyrus
ITG-l	Inferior temporal gyrus, left, lateral bank of gyrus
ITG-mts	Inferior temporal gyrus, left, bank of mts
SPL-s	Superior parietal lobule, left, superior bank of gyrus
SMG-i	Supramarginal gyrus, left, inferior bank of gyrus
PCLa-i	Paracentral lobule, anterior part, left, inferior bank of gyrus
CgGf-i	Cingulate gyrus, frontal part, left, inferior bank of gyrus
orIFG	Inferior frontal gyrus, orbital part, left
AnG-s	Angular gyrus, left, superior bank of gyrus
PHG-l	Parahippocampal gyrus, left, lateral bank of gyrus
PHG-cos	Parahippocampal gyrus, left, bank of the cos
DTLv	Lateral group of nuclei, left, ventral division
ITG-its	Inferior temporal gyrus, left, bank of the its
FuG-cos	Fusiform gyrus, left, bank of cos
SOG-s	Superior occipital gyrus, left, superior bank of gyrus

HG	Heschl's gyrus, left
PRF	Pontine reticular formation, left
Pn	Pontine nuclei, left
SNC	Substantia nigra, pars compacta, left
SNR	Substantia nigra, pars reticulata, left
RN	Red nucleus, left
VTA	Ventral tegmental area, left
DTM	medial group of nuclei, left
Sb	subthalamic nucleus, left
R	reticular nucleus of thalamus, left
ILr	rostral group of intralaminar nuclei, left
BLA	basolateral nucleus, left
LGd	dorsal lateral geniculate nucleus, left
TCd	tail of caudate nucleus, left
LA	lateral nucleus, left
COMA	cortico-medial group, left
BMA	basomedial nucleus, left
SPL-i	superior parietal lobule, left, inferior bank of gyrus
Pcu-i	precuneus, left, inferior lateral bank of gyrus
Cun-pest	cuneus, left, peristriate
Cun-str	cuneus, left, striate
GPi	globus pallidus, internal segment, left
AnG-i	angular gyrus, left, inferior bank of gyrus
SMG-s	supramarginal gyrus, left, superior bank of gyrus
GRe	gyrus rectus, left
fro	frontal operculum, left
trIFG	inferior frontal gyrus, triangular part, left
PLP	planum polare, left
PrOR	preoptic region, left
ILc	caudal group of intralaminar nuclei, left
ZI	zona incerta, left
DTLd	lateral group of nuclei, left, dorsal division
Pcu-s	precuneus, left, superior lateral bank of gyrus
cc	corpus callosum
CeA	central nucleus, left
LHM	lateral hypothalamic area, mammillary region, left
PHA	posterior hypothalamic area, left

The 105 regions shown in Supplemental Figure S3, with acronyms used in the figure and the full region names.

Supplemental Figures

Supplemental Figure 1: Gene expression compared between male and female brains

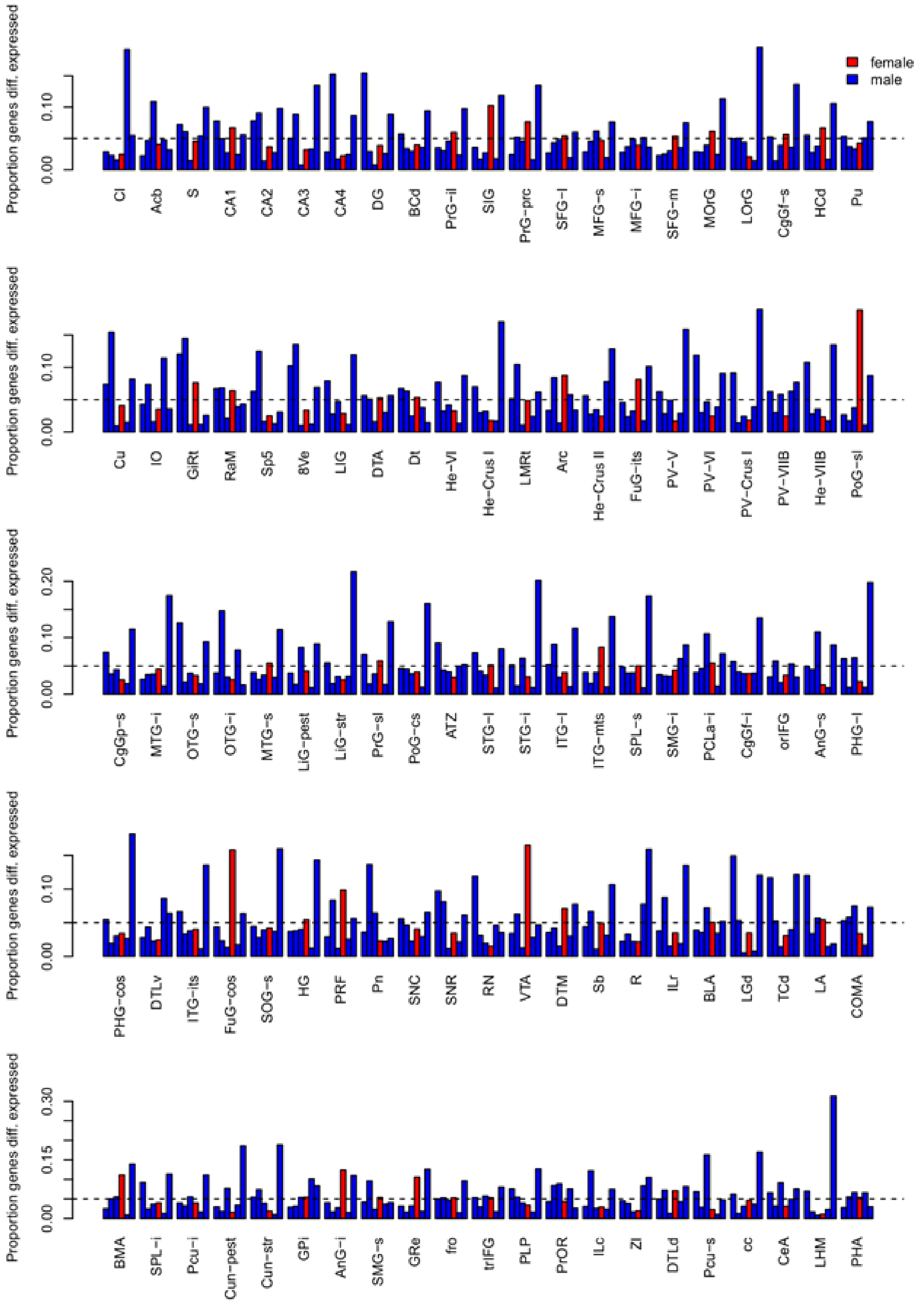

Fig. S1. The proportion of genes that is differentially expressed between donors is shown per brain region. All sample expressions were averaged to the 105 regions (indicated by the abbreviations; full names are given in Table S8 and region details at http://human.brain-map.org) present in all six brains. The bars for the female donor are shown in red, for the five male donors in blue. Because, for the given cut-off, 5% of genes are expected to be differentially expressed, a horizontal dashed line is drawn at this value. All donors have a somewhat higher proportion of differentially expressed genes in some of the brain regions, but the difference between the female brain and all male brains is, on this scale, not larger than between a male brain and all other brains.

Supplemental Figure 2. Enrichment and overlap of GO term and pathway enrichments in the 18 modules

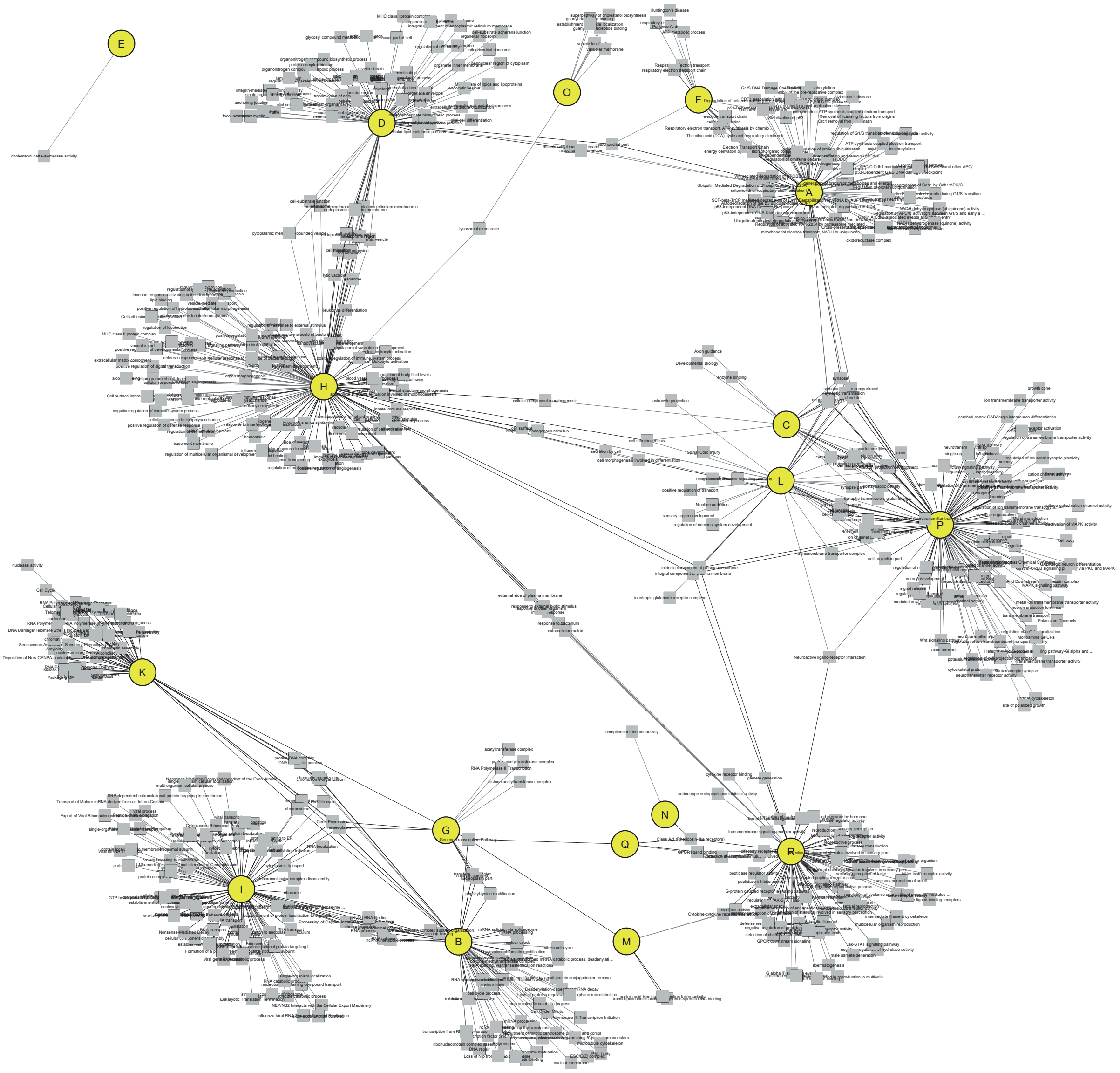

Fig. S2. GO term and pathway enrichments in the 18 modules and clustering of the results was performed with ToppCluster and visualized in Cytoscape. Modules are shown as yellow circles in the network. GO terms are shown as grey squares and are connected to the modules if enriched in that module. GO terms shared between modules are only shown once, and are connected to all modules enriched for the GO term.

Supplemental Figure 3. Brain location overview in the coronal brain slices

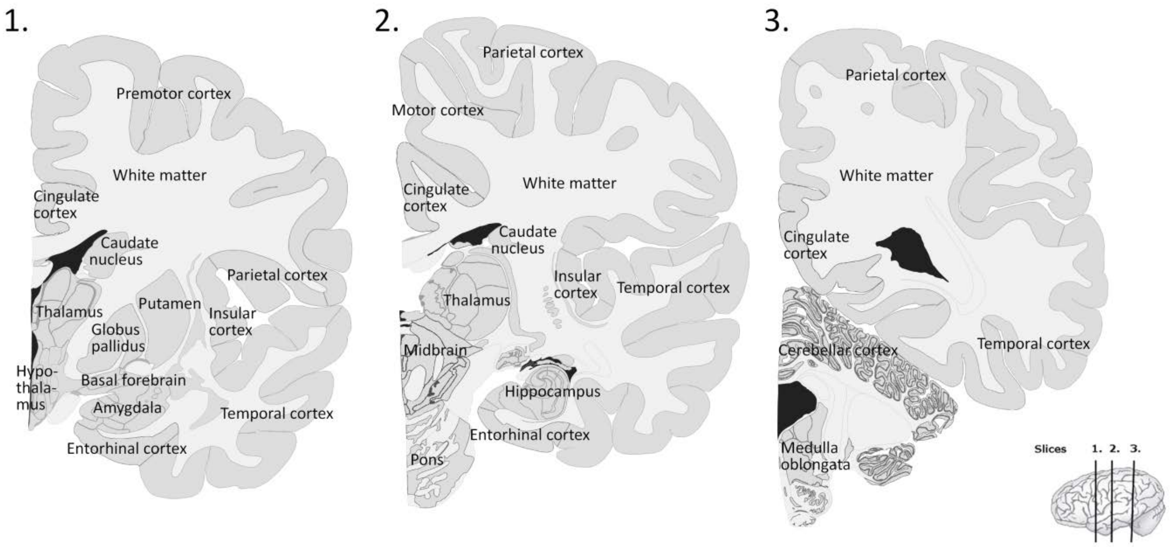

Fig. S3. The three coronal human brain slices (1-3) from three different locations in the brain (legend) visualize the regions with gene expression data in the Allen Human Brian Atlas. Ventricles are shown in black. These slices are used in Figure 1-3 to visualize the brain regions and gene expression patterns.

Supplemental Figure 4: Selection of cut-off threshold value for co-expression analyses

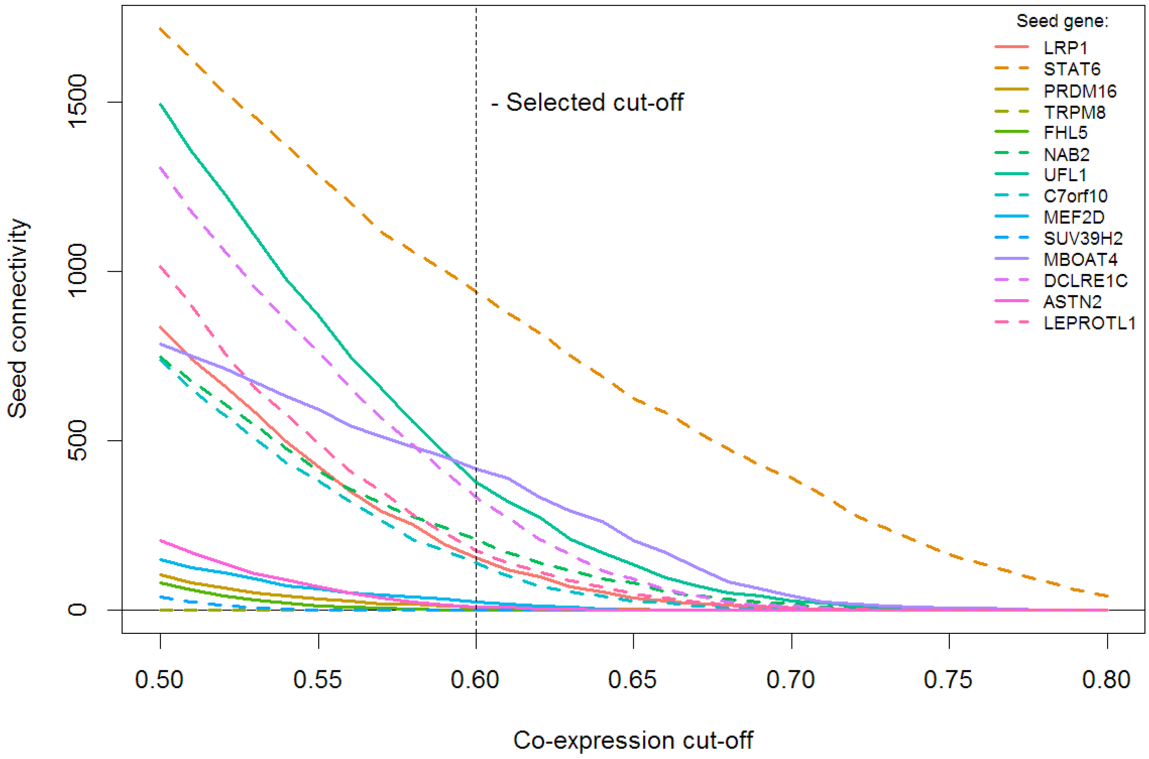

Fig. S4. The connectivity (number of connections) of each high confidence seed gene in the local co-expression network, for a range of threshold levels (co-expression cut-off). At a threshold value of 0.8 only the most connected gene (STAT6) will have connections. We selected the cut-off of 0.6, because at this value STAT6 has less than 1000 connections and most of the other genes retain a number of connections.

Supplemental References

Langfelder P and Horvath S (2008) WGCNA: an R package for weighted correlation network analysis. BMC Bioinformatics 9: 559.

Li M-X, Yeung JMY, Cherny SS, Sham PC (2012) Evaluating the effective numbers of independent tests and significant p-value thresholds in commercial genotyping arrays and public imputation reference datasets. Human Genetics 131: 747–56.

Murtagh F and Legendre P (2014) Ward's Hierarchical Agglomerative Clustering Method: Which Algorithms Implement Ward's Criterion? Journal of Classification 31: 274–295.

R Core Team (2014) R: A Language and Environment for Statistical Computing. Vienna, Austria. Retrieved from www.r-project.org
